# Apparent Diffusion Coefficient (ADC) Histogram Analysis in Parotid Gland Tumors: Evaluating a Novel Approach for Differentiation between Benign and Malignant Parotid Lesions Based on Full Histogram Distributions

**DOI:** 10.3390/diagnostics12081860

**Published:** 2022-08-01

**Authors:** Tobias Hepp, Wolfgang Wuest, Rafael Heiss, Matthias Stefan May, Markus Kopp, Matthias Wetzl, Christoph Treutlein, Michael Uder, Marco Wiesmueller

**Affiliations:** 1 Institute of Medical Informatics, Biometry and Epidemiology, Friedrich-Alexander-Universität Erlangen-Nürnberg (FAU), 91054 Erlangen, Germany; tbs.hepp@fau.de; 2 Institute of Radiology, University Hospital Erlangen, Friedrich-Alexander-Universität Erlangen-Nürnberg (FAU), 91054 Erlangen, Germany; wolfgang.wuest@martha-maria.de (W.W.); rafael.heiss@uk-erlangen.de (R.H.); matthias.may@uk-erlangen.de (M.S.M.); markus.kopp@uk-erlangen.de (M.K.); matthias.wetzl@uk-erlangen.de (M.W.); christoph.treutlein@uk-erlangen.de (C.T.); michael.uder@uk-erlangen.de (M.U.); 3 Imaging Science Institute, University-Hospital-Erlangen, Friedrich-Alexander-Universität Erlangen-Nürnberg (FAU), 91054 Erlangen, Germany

**Keywords:** apparent diffusion coefficient, head and neck MRI, multimodal imaging, parotid gland tumor, histogram analysis, cross-validation techniques

## Abstract

The aim of this study was to assess the diagnostic value of ADC distribution curves for differentiation between benign and malignant parotid gland tumors and to compare with mean ADC values. 73 patients with parotid gland tumors underwent head-and-neck MRI on a 1.5 Tesla scanner prior to surgery and histograms of ADC values were extracted. Histopathological results served as a reference standard for further analysis. ADC histograms were evaluated by comparing their similarity to a reference distribution using Chi^2^-test-statistics. The assumed reference distribution for benign and malignant parotid gland lesions was calculated after pooling the entire ADC data. In addition, mean ADC values were determined. For both methods, we calculated and compared the sensitivity and specificity between benign and malignant parotid gland tumors and three subgroups (pleomorphic adenoma, Warthin tumor, and malignant lesions), respectively. Moreover, we performed cross-validation (CV) techniques to estimate the predictive performance between ADC distributions and mean values. Histopathological results revealed 30 pleomorphic adenomas, 22 Warthin tumors, and 21 malignant tumors. ADC histogram distribution yielded a better specificity for detection of benign parotid gland lesions (ADC_histogram_: 75.0% vs. ADC_mean_: 71.2%), but mean ADC values provided a higher sensitivity (ADC_mean_: 71.4% vs. ADC_histogram_: 61.9%). The discrepancies are most pronounced in the differentiation between malignant and Warthin tumors (sensitivity ADC_mean_: 76.2% vs. ADC_histogram_: 61.9%; specificity ADC_histogram_: 81.8% vs. ADC_mean_: 68.2%). Using CV techniques, ADC distribution revealed consistently better accuracy to differentiate benign from malignant lesions (“leave-one-out CV” accuracy ADC_histogram_: 71.2% vs. ADC_mean_: 67.1%). ADC histogram analysis using full distribution curves is a promising new approach for differentiation between primary benign and malignant parotid gland tumors, especially with respect to the advantage in predictive performance based on CV techniques.

## 1. Introduction

Although salivary gland tumors account only for approximately 3–6% of all head and neck tumors, the majority of these lesions are located within the parotid gland [1,2]. Primary parotid gland tumors offer a wide variety of histological types and subtypes, predominantly benign lesions [3,4]. The most common benign parotid gland tumors are pleomorphic adenomas (PA) and Warthin tumors (WT) [5,6,7]. However, less frequently encountered, malignant parotid gland tumors (MT), e.g., adenoid cystic carcinoma or mucoepidermoid carcinoma usually require extensive surgery, often accompanied with the loss of the facial nerve [8,9,10,11]. Clinically, benign and malignant parotid gland tumors are hard to distinguish from each other and the tumor extension may be underestimated [12,13]. Therefore, a reliable imaging modality is essential for sufficient preoperative assessment [14,15]. Ultrasound (US) is widely available and plays an important role in initial assessment of parotid gland tumors, but is limited to superficial neck regions [16,17,18,19]. Magnetic Resonance Imaging (MRI) is commonly used in clinical routine to assess parotid gland tumors with regard to their full extension and potential infiltration of adjacent structures. Conventional MRI sequences exhibit basic tumor characteristics, e.g., tumor margins, heterogeneity, and infiltration of adjacent structures [14]. Despite its advantages for the assessment of tumor extension and potential infiltration, its role for precise differentiation between benign and malignant entities may be limited and therefore controversial [20,21].

The value of functional MRI techniques in parotid gland tumor diagnostics has been shown in the literature and may contribute to a deeper insight into tumor biology [22,23,24]. Alongside perfusion characteristics, diffusion-weighted imaging (DWI), and the derived apparent diffusion coefficient (ADC) value are frequently used to characterize parotid gland tumors [25,26]. DWI and its derived ADC value provide quantitative information about the Brownian motion of water molecules with respect to its cellular environment [27]. Restricted diffusion is typically observed in tissues with increased cellularity and decreased interstitial space, thus leading to low ADC values [27]. Although in theory, malignant tumors usually exhibit lower ADC values than benign tumors, Habermann et al. observed an overlap of mean ADC values between malignant and benign parotid gland tumors [28]. Alternative ADC parameters, such as median, different percentiles, and histogram skewness were evaluated, and some were found to be useful [29,30,31]. Nevertheless, to the best of our knowledge, no study exists evaluating the whole histogram distribution of ADC values in benign and malignant parotid gland tumors.

Therefore, the aim of this study was to evaluate the diagnostic performance of ADC histogram distribution analysis for differentiating between malignant and benign parotid gland tumors.

## 2. Materials and Methods

This prospective study was conducted in accordance with the guidelines of the Declaration of Helsinki and approved by the Institutional Review Board of Friedrich-Alexander Universität Erlangen/Nürnberg.

### 2.1. Study Population and Study Procedure

Based on the clinical assessment by a consultant physician for otorhinolaryngology, only patients with a newly suspected parotid gland tumor were prospectively included in this study. In total, 81 patients underwent MRI for pre-operative assessment. Patients with contraindications for MRI examination (such as a pacemaker, metal fragments, unsuitable implants or claustrophobia) were excluded. Furthermore, patients with insufficient or absent DWI, biopsy prior to MRI, and a tumor volume below 1 cm^3^ were also excluded. All patients underwent surgery after MRI examination and histopathological findings served as a reference standard.

### 2.2. Imaging Technique

All MRI examinations were performed on a 1.5 Tesla (T) MRI scanner (MAGNETOM Aera, Siemens Healthcare GmbH, Erlangen, Germany) with a dedicated 20-channel head and neck coil using a routine examination protocol for parotid gland tumors with DWI sequence as stated below. Our routine examination protocol for parotid gland tumors consists of a native T1-weighted sequence in axial slice orientation with 3 mm slice thickness, a native T2-weighted sequence in axial slice orientation with 3 mm slice thickness, a native T2-weighted Short-TI-Inversion-Recovery (STIR) sequence in axial and coronal slice orientation, each with 3 mm slice thickness, and a post-contrast T1-weighted sequence with spectral fat saturation in axial and coronal slice orientation, each with 3 mm slice thickness.

An echo-planar DWI sequence was measured in axial slice orientation using three *b*-values (0, 500, and 1000 s/mm^2^) in three orthogonal directions (detailed sequence parameters are listed in Table 1). Based on the DWI data, ADC values were calculated in-line on the scanner console and were displayed as parametric maps in axial slice orientation with 5 mm slice thickness.

### 2.3. Image Analysis and ADC Measurement

The image analysis and ADC measurements were performed by one senior radiologist with 10 years of experience in head and neck MRI. Further clinical information or other imaging data was not available to the reader.

**Figure 1:**
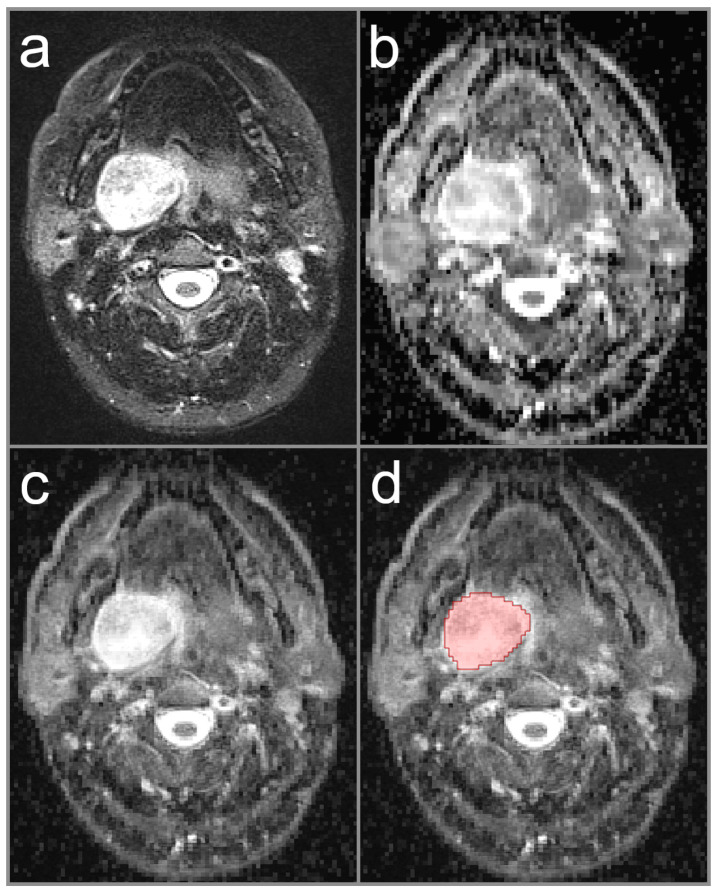
Image illustrating a study patient with pleomorphic adenoma in the deep lobe of the right parotid gland (red area indicates lesion on this particular slice). The image on the left upper row (**a**) shows the T2-weighted image with fat saturation, clearly delineating the tumor boundaries. The image on the right upper row (**b**) displays the parametric ADC map. The image on the left lower row (**c**) illustrates the co-registration of the ADC map and T2-weighted image with a mix ratio of 50%. The image on the right lower row (**d**) shows the co-registered image with the region-of-interest illustrated.

**Figure 2:**
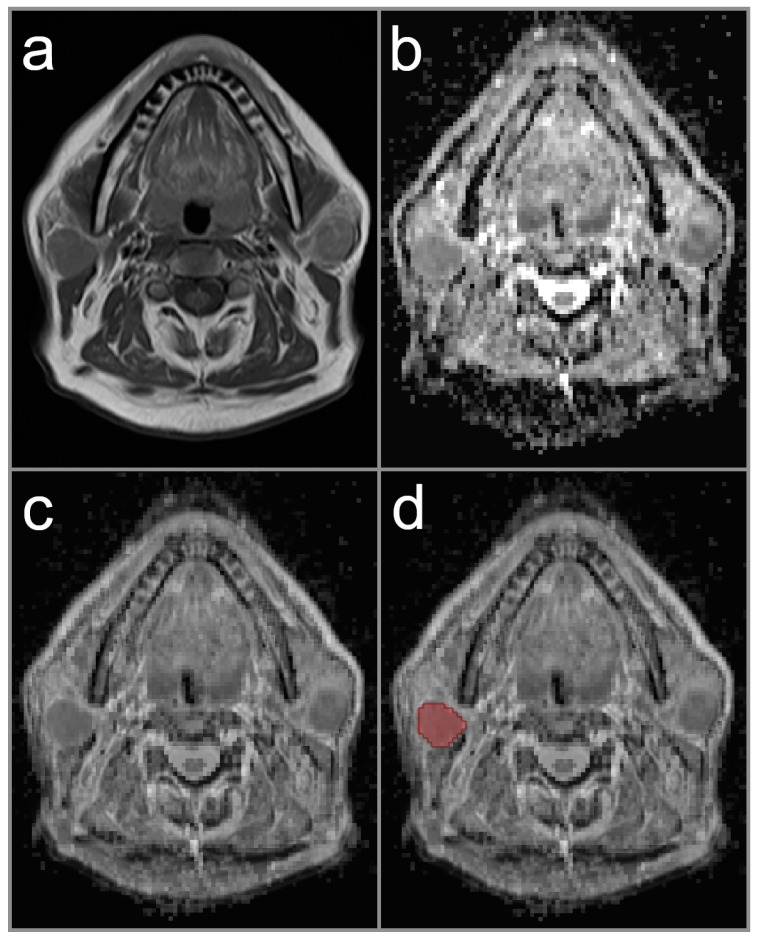
Image illustrating a study patient with bilateral Warthin tumors (red area indicates lesion on this particular slice). The image on the left upper row (**a**) displays native T1-weighted image with hypointense signal intensity of the tumors. The image on the right upper row (**b**) shows the parametric ADC map, illustrating the characteristic low ADC values of Warthin tumor. The image on the left lower row (**c**) represents the co-registration of the ADC map and T1-weighted image with a mix ratio of 50 %. The image on the right lower row (**d**) illustrates the co-registered image with the region-of-interest illustrated on the right side. The right parotid lesion was chosen due to its bigger size.

**Figure 3:**
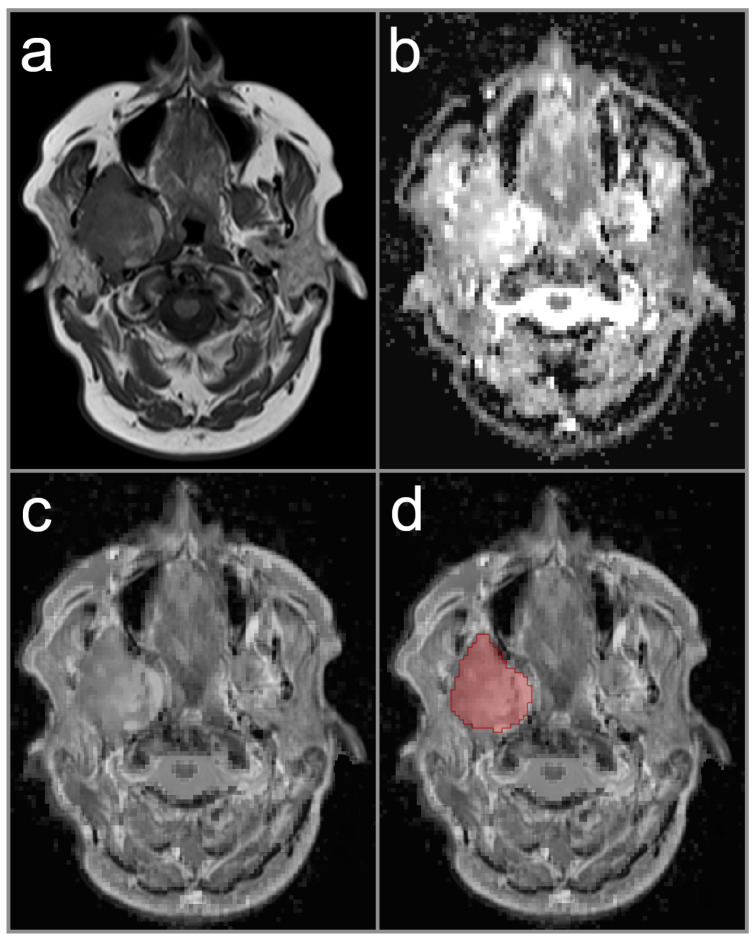
Image illustration a study patient with mucoepidermoid carcinoma in the deep lobe of the right parotid gland (red area indicates lesion on this particular slice). The image on the left upper row (**a**) shows native T1-weighted image with predominantly hypointense signal intensity of the parotid lesion. The image on the right upper row (**b**) represents the parametric ADC map. The image on the left lower row (**c**) shows the co-registration of the ADC map and T1-weighted image with a mix ratio of 50 %. The image on the right lower row (**d**) displays the co-registered image with the region-of-interest illustrated.

ADC data was processed using a dedicated software tool (Medical Imaging Interaction Toolkit (MITK), v2018.04.2, German Cancer Research Center (dkfz), Heidelberg, Germany) to measure the ADC values in the whole tumor volume (Volume-of-Interest, VOI). During VOI placement, co-registration of native T1-weighted, post-contrast T1-weighted, or T2-weighted MRI sequences were used for exact delineation of tumor boundaries on each image slice in axial orientation. The reader decided which conventional MRI sequence was appropriate for co-registration in each study case. Each drawn VOI in the ADC data was checked for consistency with co-registered conventional MRI sequence to avoid partial volume effects. In cases of multiple parotid lesions, the largest lesion was chosen for ADC assessment. Representative cases are provided in Figure 1, Figure 2 and Figure 3.

### 2.4. Statistical Analysis

**Figure 4:**
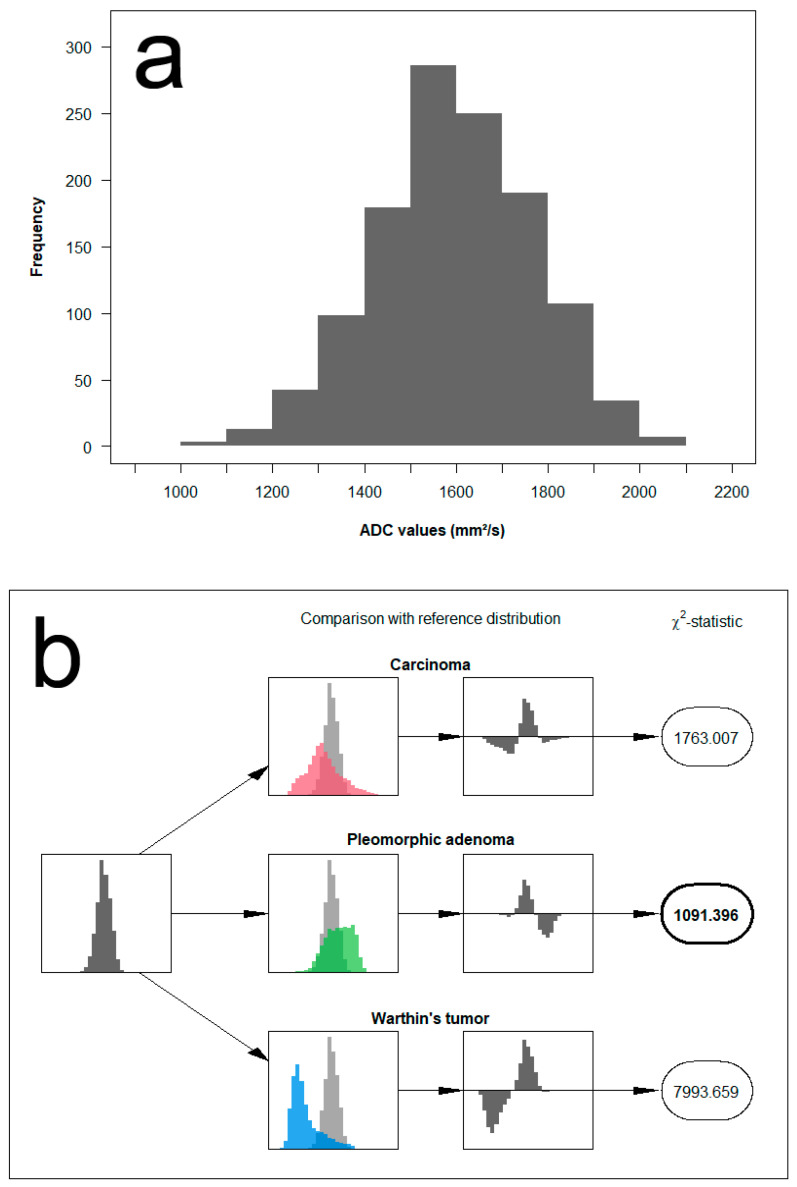
Example of histogram distribution derived from the patient of Figure 1. The image on the upper row (**a**) shows the full distribution of ADC values of a pleomorphic adenoma. The image on the lower row (**b**) illustrates the analysis algorithm containing a comparison of the measured ADC distribution to each of the three reference distributions using a Chi^2^ test-statistic. In this example case, the test-statistic clearly suggests a match with the reference distribution of pleomorphic adenomas.

**Figure 5:**
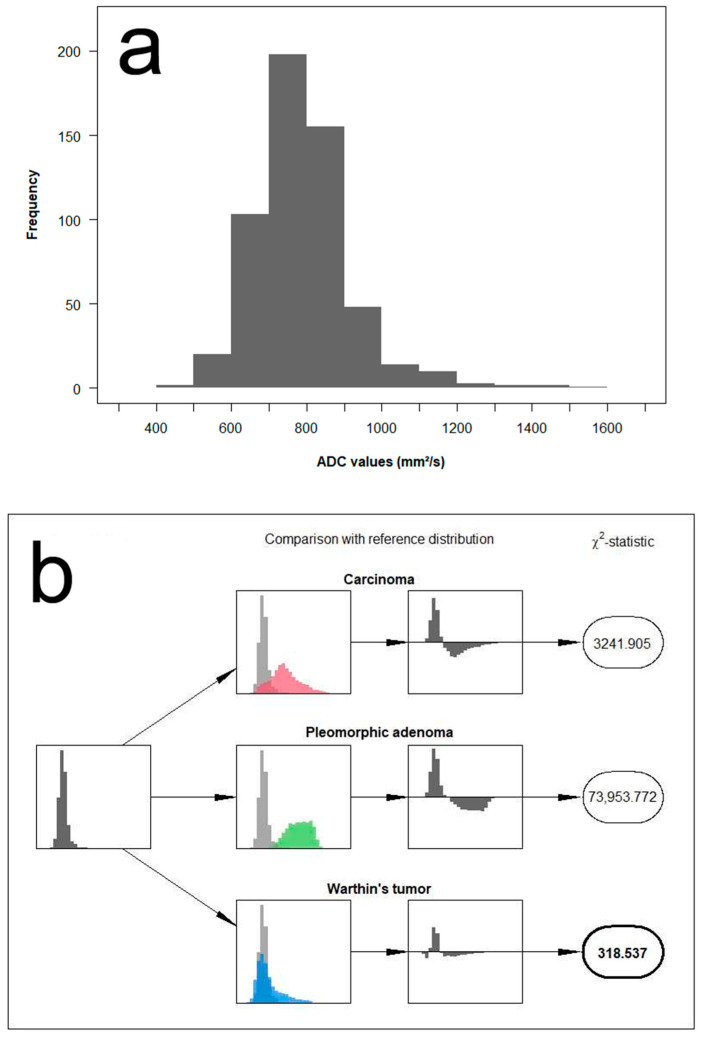
Example of histogram distribution derived from the patient of Figure 2. The image on the upper row (**a**) represents the full distribution of ADC values of a Warthin tumor. The image on the lower row (**b**) illustrates the analysis algorithm compromising a comparison of the measured ADC distribution to each of the three reference distributions using a Chi^2^ test-statistic. In this example case, the test-statistic assumes a match with the reference distribution of Warthin tumors.

**Figure 6:**
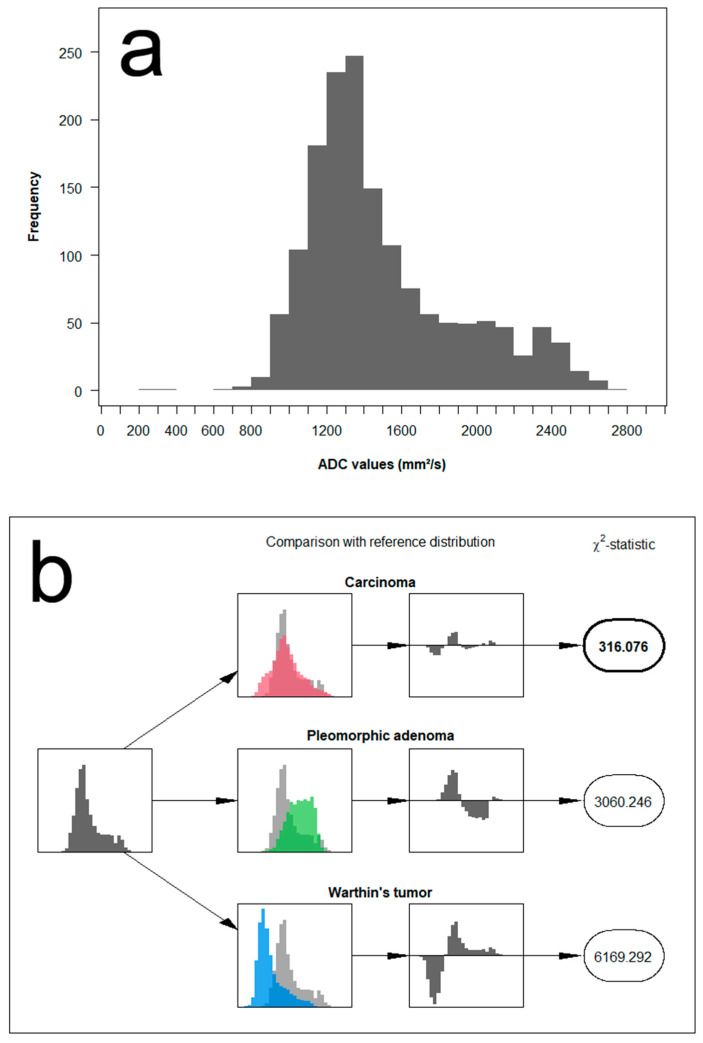
Example of histogram distribution derived from the patient of Figure 3. The image on the upper row (**a**) illustrates the full distribution of ADC values of a malignant parotid lesion. The image on the lower row (**b**) shows the analysis algorithm including a comparison of the measured ADC values to each of the three reference distributions using a Chi^2^ test-statistic. In this example case, the test-statistic indicates a match with reference distribution of malignant parotid tumors.

We propose a new approach that makes use of the full distribution of measured ADC values for the differentiation between the different parotid tumors. The concept is based on comparing the distribution of ADC values measured in a single patient with reference distributions and assessing the similarity to each other. Example cases illustrating the analysis algorithm are provided in Figure 4, Figure 5 and Figure 6.

In detail, the method is applied and evaluated as follows: First, we categorized the range of measurements into fixed ADC intervals of 100 mm^2^/s and grouped the measurements of all patients accordingly. Based on the histopathological results, we classified parotid gland tumors into three groups: pleomorphic adenoma, Warthin tumor, and malignant tumors. Next, we pooled the data from each of the three lesion categories to generate the empirical probability mass function, representing its corresponding reference distribution. As a result, this allows the computation of Chi^2^ test-statistics $T_{t}^{\left(i\right)}$ for each patient *i* and tumor type *t* from

$$T_{t}^{\left(i\right)}=\sum_{m=1}^{M}\frac{{\left(O_{m}^{\left(i\right)}-E_{t,m}\right)}^{2}}{E_{t,m}},$$

where $O_{m}^{\left(i\right)}$ denotes the number of measurements for patient *i* in the *m*-th interval and $E_{t,m}$, the expected number of measurements in the same interval if the patients’ measurements were to come from the reference distribution of tumor type *t*. The final decision is then based on the magnitude of the resulting test-statistic, with the smallest $T_{t}^{\left(i\right)}$ indicating the most likely of the three reference distributions.

Moreover, we computed diagnostic thresholds for the individual mean ADC values from our data in order to provide a proper comparison to a commonly used approach in clinical routines. Thresholds were chosen so that they maximize the Youden Index in the comparison between either MT and PA or MT and WT. We calculated sensitivity and specificity for both methods regarding their ability to differentiate between malignant and benign tumors.

In addition, we provided estimates for the predictive performance using three different cross-validation techniques. The idea behind this strategy was to construct a reference distribution and threshold values by using only a fraction of the full data, i.e., the training data. The evaluation of correctly classified tumor entities was then performed on the remaining test cases that were not used in the calibration process of the diagnostic classifiers and therefore did not directly influence it, as would also be the case with future patient data. To be specific, we used leave-one-out CV, repeated (10-fold) CV, and bootstrapping with 1000 sampling iterations for the latter two.

All analyses were performed using the statistical software environment R (R Core Team (2021). R: A language and environment for statistical computing. R Foundation for Statistical Computing, Vienna, Austria).

## 3. Results

### 3.1. Patient Population

In total, 73 data sets were eligible for further analysis (the following exclusion criteria were applied: 2 patients with insufficient DWI data, 3 patients with biopsy prior to MRI, and 3 patients with tumor volume below 1 cm^3^). The study population consisted of 35 female (48%) and 38 male (52%) patients. Mean age was 66 ± 18 years (range: 26–93 years). Thirty patients (41%) were finally diagnosed with pleomorphic adenoma after surgical excision and subsequent histopathological confirmation. Twenty-two patients (30%) were diagnosed with Warthin tumor and twenty-one patients (29%) with malignant tumor. Table 2 provides detailed information about the composition of malignant parotid lesions evaluated in this study.

### 3.2. Diagnostic Performance

ADC histogram distribution yielded a better specificity for detection of benign parotid gland lesions (ADC_histogram_: 75.0% vs. ADC_mean_: 71.2%), but mean values provided a higher sensitivity (ADC_mean_: 71.4% vs. ADC_histogram_: 61.9%). By contrast, a perfect sensitivity was found for ADC distribution to distinguish MT from PA (ADC_histogram_: 100% vs. ADC_mean_: 95.2%), whereas the specificity was slightly higher for mean values (ADC_mean_: 73.3% vs. ADC_histogram_: 70.0%). A higher sensitivity was found for mean values to distinguish MT from WT (ADC_mean_: 76.2% vs. ADC_histogram_: 61.9%), but ADC distribution demonstrated an increased specificity regarding this subgroup (ADC_histogram_: 81.8% vs. ADC_mean_: 68.2%). Table 3 provides a detailed overview of sensitivity and specificity results.

### 3.3. Cross-Validation Performance

In general, ADC distributional analysis exhibited a higher proportion of correctly classified tumor entities compared to mean ADC values for all three CV techniques. A detailed overview is provided below and summarized in Table 4.

Full histogram analysis consistently performs better in terms of predictive total accuracy, indicating a relatively lower generalization error as, unlike the values reported in Table 3, the evaluated cases are not used to generate the thresholds and reference distributions. The same applies to the type-specific precision, meaning that compared to the approach using only the individual average of the ADC values, a higher proportion of a specific predicted type is actually correct. In contrast, the true positive rate is the proportion of correct predictions with respect to a specific true type in the test sets and could therefore be interpreted as “test set sensitivity”. Here, the picture is more balanced but still slightly in favor of the full histogram analysis.

## 4. Discussion

In this study, we evaluated a new method to differentiate between benign and malignant parotid lesions based on the whole histogram of the ADC values by comparing a specific histogram distribution to previously specified reference distributions and evaluating their similarity. The method is compared with the common practice of differentiating via thresholds using mean ADC values. ADC histogram distributions yielded a better specificity for the detection of benign parotid gland tumors, whereas the use of mean ADC values demonstrated a higher sensitivity.

Due to its high soft-tissue contrast and the possibility to combine morphological information with functional techniques, in particular DWI and dynamic contrast-enhanced (DCE) imaging, multiparametric MRI represents the modality of choice in patients with parotid gland tumors [23,24]. Accurate differentiation between benign and malignant parotid lesions are crucial for the determination of different therapeutic strategies and might be a predictor of disease prognosis [32,33,34]. DWI used in parotid diagnostics is a very popular tool and its qualities as a non-invasive biomarker are studied in various articles in recent years [20,26,35]. Nevertheless, methodical and technical issues may limit the diagnostic value of DWI and its derived ADC values: Region-of-interests for ADC measurements are commonly drawn on a single slice, whereas encompassing the whole lesion is a time-consuming process and therefore often not feasible in clinical routine. Although a review article by Bruvo et al. described no significant difference in mean ADC values of the parotid gland parenchyma between single-slice and whole lesion ADC assessment, single slice ROIs cannot fully display tissue heterogeneity and its intra- and interreader agreement may be reduced [36]. In this study, we used the whole lesion assessment approach to include a maximum of information derived from each parotid lesion for further analysis. A recent study by Zhang et al. emphasized the usefulness of whole lesion ADC measurements for differentiating benign and malignant parotid tumors in combination with histogram analysis [31]. One of their investigated parameters was histogram skewness, a measure of asymmetry of the probability distribution. For example, malignant parotid tumors exhibit a positive (right) skewness, which is based on a majority of dense packed cells (low ADC values) and a minority of scattered necrotic areas (high ADC values). In contrast, PA mostly showed a negative (left) skewness, thus skewness might be an additional biomarker for tumor biology and its heterogeneity. Therefore, whole lesion measurements might capture additional information for precise tumor classification and might become a standard procedure in parotid lesion assessment, whereas single-slice measurements may leave out important information by chance. Future developments, like deep learning-based automated algorithms for whole lesion segmentation, may facilitate the implementation in clinical routine [37].

Beside the above-discussed methods of ADC data collection, choosing the appropriate ADC parameter for clinical decision-making is highly crucial. Mean ADC values are commonly used in clinical routine, since mean values are easy and quick to obtain, and their interpretation is straightforward [20]. Nevertheless, mean ADC values do not represent a robust parameter for the differentiation between benign and malignant parotid lesions, since their range of values is reported to possibly overlap [28]. In recent studies, different alternative ADC parameters were proposed for parotid lesion assessment, e.g., median and various percentiles [29]. Ma et al. showed superior diagnostic performance by using the 10th ADC percentile in comparison to mean ADC values [30]. They assumed that low-percentile ADC values are more likely to represent tumor areas with high cell density, thus possibly indicating a more aggressive biological behavior. By contrast, we used the whole histogram distribution for tumor differentiation and observed a consistent increase in diagnostic performance with respect to the cross-validated prediction accuracy. While these findings indicate an advantage using the full information of the shape and distribution of ADC values, the success of the proposed method strongly depends on the quality of the reference distributions, which have to be estimated beforehand. In this context, we divided the benign tumor group into two subgroups, PA and WT, to provide a dedicated subgroup analysis since these entities represent the most frequent tumor types in the parotid gland, and more importantly to obtain two separate reference distributions based on the fact that, PA and WT usually manifest with opposite ADC values, with PA usually higher and WT lower [26]. Blending these contrastive spectra into a single reference distribution would lead to higher Chi^2^-statistics as the data will only fit one or the other part of the mixture and therefore, likely resulting in misdiagnosis. In fact, this is the same reason why two separate thresholds are adopted in the traditional comparison of mean ADC values to which we compare our results. Naturally, a larger sample would provide better estimates for the three reference distributions used in this study, thus would possibly help to consolidate our results. In addition, pooled data from multiple centers may help to establish a standardized reference distribution among different tumor entities and their subtypes. This aspect seems to be desirable as malignant parotid tumors are rare compared to other malignancies in the head and neck area, thus relatively scarce encountered in a single center. Compared to the threshold values for the mean ADC, manually calculating the Chi^2^ test-statistics also involves slightly more effort. With larger samples, it might therefore make sense to describe the reference distribution using parametric models in order to reduce the number of parameters. The Chi^2^-test-statistic could then be possibly replaced by the sums of the likelihoods of the ADC values, but the general concept remains the same.

Technical considerations are another important aspect when discussing different ADC parameters and their comparison. Changes in the DWI sequence parameters may result in significant changes on ADC histogram; thus, sequence parameters should be kept constant. The choice of different *b*-values can significantly affect the ADC calculation [38]. Using low *b*-values < 300 s/mm^2^ is likely to result in higher ADC values, by contrast, the use of high *b*-values in absence of b = 0 s/mm^2^ presumably leads to lower ADC values. In our study, we measured three *b*-values (0, 500 and 1000 s/mm^2^), which seems to be in good correlation with other studies using the same range [36]. Analogous to the above-discussed methodical considerations, a general agreement would facilitate inter-center comparability.

Some limitations must be considered when interpreting the results of our study: First, due to the relatively small sample number and the huge variety of histological types of parotid gland lesions, a sufficient sub-group analysis of malignant entities was not possible. Moreover, larger sample sizes would also allow to reserve a designated test set, which is used exclusively for predictive evaluation.

Second, we only were able to perform this study on a 1.5 T MRI scanner, diagnostic performance among other magnetic field strengths remains unclear.

Third, other functional MRI techniques, such as DCE or Intravoxel Incoherent Motion (IVIM) were not included [39]. The implementation of our approach within multiparametric MRI might exhibit further progress and higher diagnostic accuracy.

## 5. Conclusions

ADC histogram analysis using full distribution curves is a promising new approach for differentiation between benign and malignant parotid gland tumors, especially with respect to the advantage in predictive performance based on cross-validation techniques.

## Figures and Tables

**Table 1:** DWI sequence parameters.

Sequence Type	Echo-Planar DWI
Repetition time [ms]	1700
Echo time [ms]	87
Voxel size [mm^3^]	2.0 × 2.0 × 5.0
Field of view [mm^2^]	250
Field of view in phase direction	100%
Phase direction	Anterior-posterior
Phase resolution	92%
Partial Fourier	75% (phase)
Matrix	128 × 128
Slice distance	20%
No. of slices	12
Parallel imaging	GRAPPA × 2
Bandwidth [Hz/pixel]	1302
Echo spacing [ms]	0.87
Readout segments	1
Flip angle [°]	180
b-values [s/mm^2^]	0, 500, 1000
Averages	6 per *b*-value
Diffusion mode	3-scan trace
Diffusion scheme	Bipolar
Acquisition time [min]	1:17

**Table 2:** Overview of malignant parotid lesions.

Pathological Result	Frequency
Mucoepidermoid carcinoma	4
Acinic cell carcinoma	6
Squamous carcinoma	2
Adenoid cystic carcinoma	3
Carcinoma ex pleomorphic adenoma	3
Ductal carcinoma	2
Merkel cell carcinoma	1

**Table 3:** Sensitivity and specificity results (bold numbers indicate superior performance).

	MT versus Benign Lesions		MT versus PA		MT versus WT	
	ADC_histogram_	ADC_mean_	ADC_histogram_	ADC_mean_	ADC_histogram_	ADC_mean_
Sensitivity	61.9%	**71.4%**	**100%**	95.2%	61.9%	**76.2%**
Specificity	**75%**	71.2%	70%	**73.3%**	**81.8%**	68.2%

**Table 4:** Cross-validation results with total accuracy giving the overall proportion of correct classifications as well as type-specific precision, i.e., the proportion of true positives from the set of corresponding predictions, and true positive rate, i.e., the proportion of true positives given the corresponding true types (bold numbers indicate superior performance).

		Leave-1-Out CV		Repeated CV		Bootstrap	
		ADC_histogram_	ADC_mean_	ADC_histogram_	ADC_mean_	ADC_histogram_	ADC_mean_
Total accuracy		**71.2%**	67.1%	**68.6%**	64.3%	**66.5%**	64.0%
Type-specific precision	MT	**50.0%**	44.4%	**45.8%**	39.4%	**44.7%**	41.0%
	PA	**100%**	91.7%	**98.5%**	89.7%	**93.0%**	82.8%
	WT	**69.2%**	68.2%	**67.2%**	65.0%	**64.1%**	62.8%
	PA|WT	**83.0%**	80.4%	**80.2%**	76.9%	**77.9%**	75.0%
Type-specific true positive rate	MT	**61.9%**	57.1%	**54.0%**	46.5%	**50.3%**	40.1%
	PA	70.0%	**73.3%**	**73.6%**	69.6%	69.4%	**75.9%**
	WT	**81.8%**	68.2%	**80.2%**	68.7%	**78.7%**	71.6%
	PA|WT	**75.0%**	71.2%	**74.4%**	71.4%	73.8%	**75.8%**

## Data Availability

All data generated and analyzed during this study are included in this published article. Raw data supporting the findings of this study are available from the corresponding author on request.
